# Unraveling of Seaweed Bioactive Substances and Their Nutritional Regulation Functions for Poultry

**DOI:** 10.3390/md23080324

**Published:** 2025-08-10

**Authors:** Si-Bing Li, Qing-Hua Yao, Xue-Qing Ye, Balamuralikrishnan Balasubramanian, Wen-Chao Liu

**Affiliations:** 1 Department of Animal Science, College of Coastal Agricultural Sciences, Guangdong Ocean University, Zhanjiang 524088, China; 11331114ff1@stu.gdou.edu.cn (S.-B.L.); yaoqinghua1@stu.gdou.edu.cn (Q.-H.Y.); yexueqing1@stu.gdou.edu.cn (X.-Q.Y.); 2 Department of Food Science and Biotechnology, College of Life Science, Sejong University, Seoul 05006, Republic of Korea

**Keywords:** seaweed, nutritional regulation, biological function, poultry production

## Abstract

Seaweed is a rich and valuable marine biological resource that contains various bioactive substances, including polysaccharides, polyphenols, fatty acids, and more. These compounds exhibit a range of biological activities, such as antimicrobial, antioxidant, and immunomodulation effects. In the face of challenges related to intensive farming, poultry are often exposed to multiple stressors during production, which can lead to oxidative stress, impaired intestinal barrier function, and excessive inflammatory responses. Due to their potent biological activities, seaweeds and their bioactive components have shown potential in improving poultry health and performance. This paper mainly reviews the classification of seaweeds and their extracts, their main biological functions, and the research progress on the application to poultry, with the aim of providing a reference for the research and application of seaweed active substances as functional feed additives in poultry.

## 1. Introduction

Seaweeds, a group of photosynthetic autotrophs widely distributed in aquatic environments, are an important component of marine biological resources. According to the report “The State of World Fisheries and Aquaculture 2024” published by the Food and Agriculture Organization of the United Nations (FAO), the global annual production of algae has reached 3.78 × 10^7^ tons in 2022. China has a coastline of approximately 32,000 km, with Guangdong Province possessing the longest coastline of about 4114.3 km, providing it with a rich endowment of seaweed resources. Based on pigment characteristics, seaweeds are primarily classified into three phyla: brown algae, red algae and green algae. Numerous studies have demonstrated that bioactive compounds extracted from seaweeds, such as polysaccharides, polyphenols, and fatty acids, possess antioxidant, antibacterial, and immunomodulatory properties [1,2,3]. Currently, the poultry industry faces multiple challenges, including the prohibition of adding antibiotics to feed [4], stress associated with intensive farming [5,6] and feed contamination by mycotoxins [7]. However, due to their abundance and diverse bioactive ingredients, seaweeds have shown promising potential in promoting healthy poultry production. For example, seaweed polysaccharides have been shown to alleviate oxidative stress and excessive inflammatory responses in heat-stressed broilers [8], red algae extracts reduce egg-yolk cholesterol [9], and green algae enhance the poultry intestinal immune barrier by modulating gut microbiota [10]. With the growing global emphasis on “antibiotic-free husbandry” and “green feed” initiatives, the application value of seaweeds and their bioactive components in the livestock and poultry industry has garnered significant scholarly attention, with research focus particularly intensifying in the field of poultry production. To date, a number of review studies have been conducted on the relationship between seaweed and poultry farming, providing a foundational basis for the development of this field. Nevertheless, existing reviews exhibit notable limitations: on the one hand, the majority of studies concentrate on single aspects, such as seaweed-processing technologies [11], their incorporation as feed ingredients in poultry diets [12], impacts on meat quality [13], or antimicrobial properties [14]; on the other hand, some studies cover a scope of livestock [15,16] and humans [17,18], and fail to conduct specific analyses tailored to poultry. Again, other existing reviews focus on the analysis of the roles of seaweed powder or a certain type of seaweed in broiler chickens [19,20]. Consequently, this review mainly discusses the classification and biological functions of seaweeds and their active substances, summarizes recent research progress on their roles in poultry nutrition regulation, and provides a more systematic theoretical framework for the application of seaweeds and their active components in poultry production.

## 2. Classification and Biological Functions of Seaweeds and Their Active Substances

**Figure 1:**
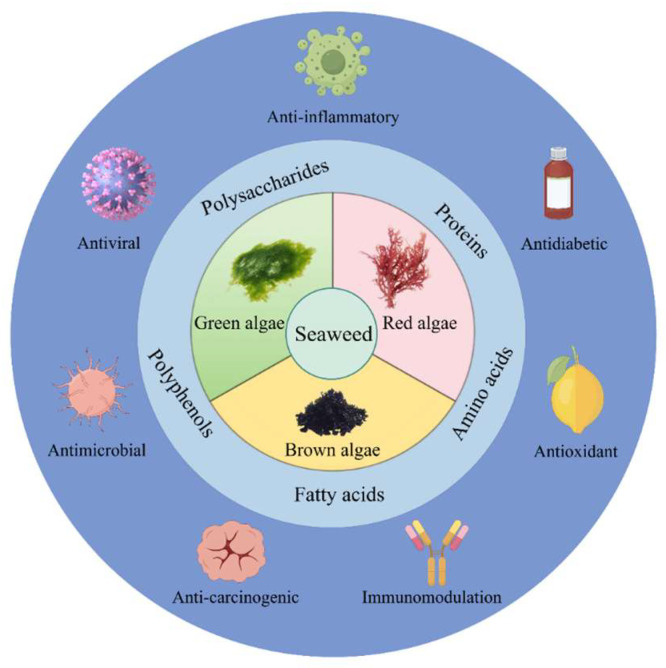
The nutritional regulation and therapeutic function of different seaweed active substances.

Seaweeds can be categorized based on the analysis of the chemical composition in Table 1. The highest protein content is found in red algae, the lowest lipid content and the highest carbohydrate content in brown algae, and the lowest dietary fiber content in green algae. In another study, it was found that the crude protein (CP) content of red algae was similar to the CP content of soybean meal-based high-protein plant feeds [21]. A study has confirmed that the active substances in seaweed have multiple biological activities, such as antibacterial, anti-inflammatory, antioxidant and immunomodulatory activities [22], which have broad application prospects in the fields of medicine, food [17,23] and agriculture [24] (Figure 1). However, in many poultry studies, seaweed, used as a feed ingredient, is susceptible to adverse effects such as high salt content and anti-nutritional factors. Various processing methods are required to eliminate these anti-nutritional factors, to optimize the use of seaweed in poultry feed [11]. Additionally, due to environmental factors, seaweed is prone to accumulating heavy metals. Therefore, when adding seaweed bioactive substances to poultry feed, it is essential to strictly control their dosage to avoid adverse effects on poultry such as bodily damage, heavy-metal accumulation, and nutritional imbalance [15,25,26].

### 2.1. Polysaccharides

**Figure 2:**
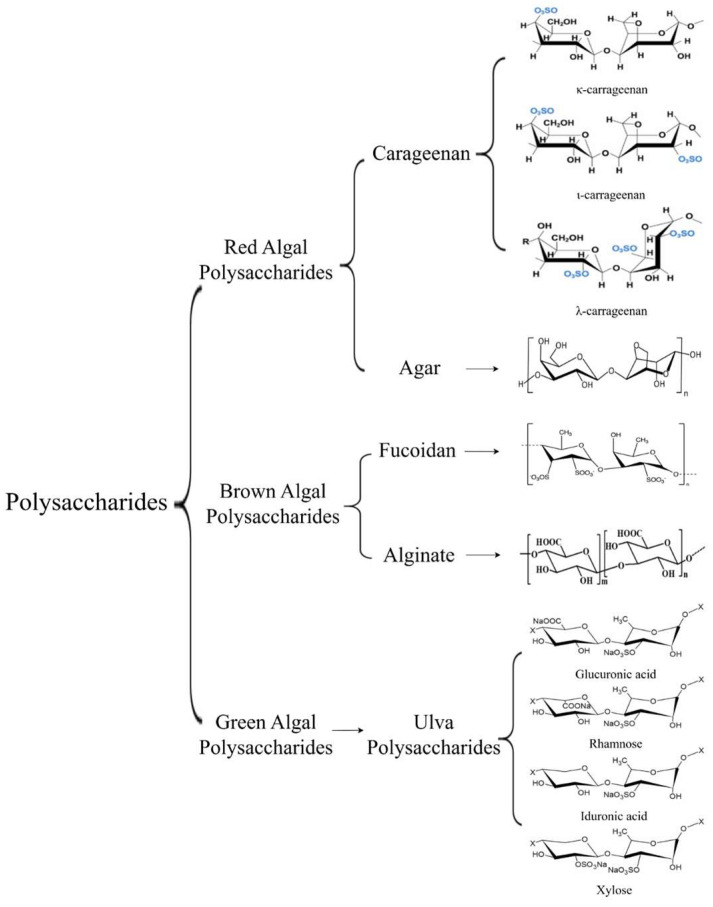
The main polysaccharide structures of seaweeds.

As shown in Figure 2, seaweed polysaccharides are macromolecular polymers consisting of glycosidic bonds, sugar chains, sulfated groups, and glucuronic acid, which have a variety of biological activities such as antioxidant, antibacterial, anti-inflammatory, and immunomodulatory (Table 2) [27,28]. At present, synthetic antioxidants [e.g., butylated hydroxytoluene (BHT)] are commonly used in China’s feed industry as an effective means of scavenging free radicals from the organism, but their potential genotoxicity (e.g., BHT-induced abnormalities in liver microsomal enzymes) limits their safety [29]. Seaweed polysaccharides exhibit excellent antioxidant activity in vivo and in vitro [30]. Seaweed polysaccharides can not only achieve antioxidant effects by directly scavenging free radicals and regulating antioxidant enzymes [e.g., superoxide dismutase (SOD), catalase (CAT)] activities, but also regulate apoptosis-related signaling pathways (e.g., Caspase-9, Bax, and JNK) to repair the oxidative damage of the organism [30,31]. In terms of immunomodulation, fucoidan can activate macrophages and regulate cytokine secretion through NF-κB and ERK/MAPK signaling pathways [32,33]. Ulva polysaccharide can effectively restore leukocyte and platelet levels in immunodeficiency models, enhance NK cell activity and remodel the anti-tumor immune microenvironment [34]. In animal experiments, feeding 1 g/kg of *Enteromorpha* polysaccharides (EP) can enhance nonspecific immunity and promote the growth and health of animals [35]. In terms of anti-inflammation, short-chain fatty acids (SCFAs) generated by microbial fermentation of seaweed polysaccharides in the intestinal tract exert a systemic anti-inflammatory effect by regulating the balance of bacterial flora and metabolism to reduce inflammatory bowel disease [36]. Meanwhile, it has been suggested that seaweed polysaccharides could alleviate the excessive inflammatory response of the organism by inhibiting the activation of the MAPK/NF-κB pathway [37].

### 2.2. Polyphenols

At the present time, the main research on polyphenols has focused on phlorotannins [45]. The phlorotannins are polymers formed by C-O or C-C bonds with m-Phloroglucinol as the basic structural unit, and their unique chemical conformation endows them with diversified biological functions [46], including antibacterial [47], anti-inflammatory [48], antioxidant [49], and anti-diabetic [50], and they belong to the category of low-toxicity chemical compounds [51]. Gallic acid and Apigenin in *Gracilaria birdiae* and *Gracilaria cornea* can scavenge DPPH free radicals and reduce reactive oxygen species (ROS) production by inhibiting NADPH oxidase [52]. The phenolic extracts from *Ascophyllum nodosum* and *Alaria esculenta* have been shown to lower blood glucose levels and inhibit the proliferation of cancer cells, respectively. These findings could provide new strategies for the treatment of diabetes and the fight against tumors [53]. Adding 600 mg/kg of phlorotannin to broiler diets can effectively alleviate liver injury. Its regulatory mechanism can be divided into two main aspects: one is to inhibit heat stress-induced iron hepatic ferroptosis by regulating the redox homeostasis of broilers [54]; the other is to mitigate Aflatoxin B1 (AFB_1_)-induced hepatic oxidative stress and mitochondrial damage by activating the Nrf2-mediated phase II detoxifying enzyme pathway (e.g., GPX1, GSTT1) and the Nrf1-regulated mitochondrial biosynthesis pathway (e.g., TFAM, MFN1) [55]. Despite the wide application of polyphenols, the instability of m-Phloroglucinol makes its extraction difficult. Novel extraction methods, such as ultrasound, microwave, pressurized liquid, pulsed electric field, supercritical fluid, and high hydrostatic pressure, can not only significantly increase the extraction rate of polyphenols, but also reduce extraction costs and minimize environmental impact [56].

### 2.3. Protein and Amino Acids

Currently, dietary proteins mainly rely on animal sources (e.g., meat, dairy products) and terrestrial plant sources (e.g., soybeans, peas). A study has shown that the CP content of brown algae (e.g., *Saccharina*) and red algae (e.g., *Gracilaria*) can reach 25–47% of the dry weight, comparable to traditional plant proteins like soybean meal (approximately 40%) and peas (approximately 25%), and their amino acid composition is balanced [57]. Moreover, seaweed as an alternative protein source in poultry nutrition can not only effectively reduce feed costs, but also improve the productivity of the poultry industry [58]. However, the digestibility of seaweed proteins in monogastric animals (e.g., poultry, swine) ranges from 40% to 60%, which is insufficient to meet the essential amino acid requirements of these animals [59]. Additionally, the nutritional and functional composition of algae varies across species, seasons, and coastal environments, making the use of seaweed proteins in poultry production challenging, in several ways [60].

### 2.4. Fatty Acids

The fatty acid composition of seaweed mainly consists of saturated fatty acids (SFAs), monounsaturated fatty acids (MUFAs) and polyunsaturated fatty acids (PUFAs). Among them, PUFA is the core component of the functional activity of seaweed lipids. The content and distribution of fatty acids are synergistically regulated by seaweed species, growth environment, and developmental stage [61]. Specifically, the eicosapentaenoic acid (EPA) content of red algae (e.g., *Porphyra*) accounted for 48.0–51.0% of the fatty acid methyl ester (FAME), while arachidonic acid and linoleic acid accounted for 2.1–10.9% and 1.3–2.5%, respectively. Brown algae (e.g., *Laminaria*) were dominated by oleic, linoleic, and α-linolenic acids, which accounted for 4.1–20.9%, 4.0–7.3% and 3.6–13.8%, respectively, and the EPA content was only 5.9–13.6% [62]. In the field of food science, seaweed fatty acids can be used as natural antioxidants, nutritional enhancers and functional food additives [63]. Fucoxanthin derived from brown algae can not only reduce obesity-related oxidative stress and inflammatory status by regulating gene expression in adipocytes, but also effectively alleviate renal aging, demonstrating anti-aging potential [64,65]. Furthermore, seaweed fatty acids have multidimensional applications in the development of pharmaceuticals and nutraceuticals, including regulation of lipid metabolism homeostasis, maintenance of blood pressure homeostasis, and anti-fatigue activity [18,66]. Research reveals that fucoxanthin improves insulin resistance and blood glucose levels through the dual mechanism of promoting energy consumption in abdominal white adipose tissue and regulating the secretion of adipokines, thus exerting anti-obesity and anti-diabetic effects [67].

## 3. Seaweeds and Their Bioactive Substances in Poultry Production

With their unique bioactive components and multi-target regulatory properties, seaweeds have become a research hotspot for functional feed additives in poultry [12,68]. Numerous studies have confirmed the ability of seaweed to synergistically enhance poultry growth performance and product quality, as well as strengthen the body’s immunity and antioxidant capacity [14,16,19,69].

### 3.1. Beneficial Effects of Seaweed Bioactive Substances in Broiler Chickens

#### 3.1.1. Improve Growth Performance

The broiler farming industry has long faced production problems [70], such as mycotoxin contamination [71], antibiotic resistance [72] and heat stress [73]. Studies have found that seaweed polysaccharides alleviate heat stress-induced bursa cell apoptosis and intestinal inflammatory responses by regulating the NF-κB signaling pathway and Th1/Th2 immune balance [74,75]. In the field of antibiotic substitution, the addition of seaweed polysaccharides can promote body weight gain, enhance immune capacity, and reduce the incidence rate in broiler chickens by regulating gene expression and influencing the activity of proteins and enzymes [10,76]. Moreover, its effect is comparable to that of antibiotic growth promoters [77]. It can be seen from Table 3 that the applied research on seaweed bioactive substances in broiler production has formed systematic achievements. Further interpretation of their core values is reflected in multiple functions such as mycotoxin detoxification, antibiotic substitution, and heat stress relief [78]. Additionally, seaweeds are also effective for other poultry, such as quails. Supplementation with 2% *Sargassum siliquastrum* increases the concentrations of total volatile fatty acids (VFAs) and propionic acid, elevates the count of *Lactobacillus* sp., and reduces the numbers of *Escherichia coli* (*E. coli*) and *Clostridium perfringens*. These effects help to regulate the cecal microbial community and fermentation characteristics, thereby enhancing antioxidant and immune functions [79]. Adding seaweeds to the poultry diet can also improve the palatability of feed and increase the feed intake of broilers [80]. For example, incorporating *Polysiphonia* sp. into feed formulations helps reduce feed hardness, which is particularly suitable for the preparation of duck feed and granular binders [81]. Although seaweeds can serve as components of broiler feed and have high nutritional value, the problem of their high ash content still needs to be addressed through pretreatment, to improve digestibility for efficient utilization [82].

#### 3.1.2. Improve Meat Quality

In addition to promoting growth performance, seaweed used as a feed additive can also enhance the meat quality of broilers [13,93]. Firstly, seaweeds can significantly modify the muscle fatty acid composition in broilers by regulating lipid metabolism pathways, increasing the content of PUFA, and thus improving meat color [94]. Secondly, as a functional additive, green algae is rich in various bioactive ingredients (e.g., proteins, polysaccharides, vitamins, etc.) and has a balanced amino acid profile, offering multiple advantages in broiler production. On the one hand, green algae can reduce heavy-metal bioaccumulation, helping to overcome barriers to commercial application [95]. On the other hand, its antioxidant constituents inhibit microbial growth in meat, delay oxidative reactions, and significantly improve various aspects of meat quality, including color, tenderness, water-holding capacity, and nutritional value, while also extending shelf life [31,95]. Although the inclusion of green algae powder in broiler diets may slightly affect the overall feed conversion ratio, it does not negatively impact blood parameters, meat quality, or product stability [96]. Therefore, green algae hold promise as a sustainable and effective feed additive for improving broiler meat quality.

#### 3.1.3. Promote Intestinal Barrier Function

**Figure 3:**
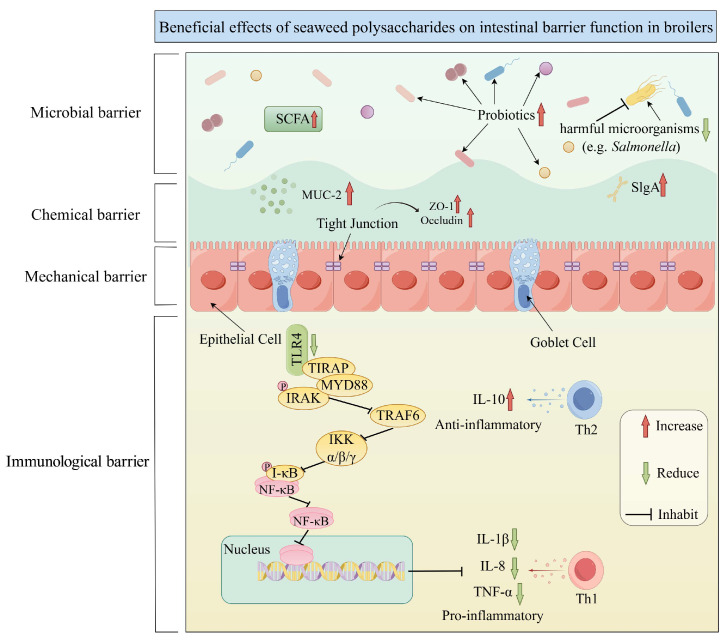
Beneficial effects of seaweed polysaccharides on intestinal barrier function in broilers.

The intestinal barrier is a core line of defense for maintaining broiler health and performance, and it plays a key role in resisting pathogen invasion, regulating nutrient absorption and maintaining intestinal homeostasis. However, *Salmonella* infection and heat stress are the main threatening factors which disrupt intestinal barrier function through multiple pathways: the former damages intestinal structure by inducing mucosal injury, inflammatory cascade reactions, and microbial flora imbalance; the latter leads to intestinal morphological abnormalities by inducing intestinal oxidative stress, inflammatory responses, and altering microbial diversity [5,97]. Against this backdrop, seaweeds, due to their unique bioactive components, provide an innovative solution for intestinal health regulation. Seaweed polysaccharides can alleviate intestinal inflammatory responses by regulating the NF-κB signaling pathway and Th1/Th2 immune balance, further promoting the intestinal immune barrier (Figure 3) [74]. Supplementation with 2000 mg/kg *Gracilaria lemaneiformis* polysaccharides can increase the content of lactic acid bacteria in the cecum, reduce the number of *E. coli*, and achieve synergistic improvement of immune barrier function [98]. Similarly, sodium alginate oligosaccharides also have the same effect [99]. In addition, dietary supplementation with brown algae powder and its extracts can enhance the abundance of beneficial microorganisms in the intestines of heat-stressed broiler chickens, improve intestinal morphology, and thus alleviate heat stress damage [100]. Supplementation of 1000–4000 mg/kg EP in poultry diets exhibits a dual protective effect: on the one hand, it significantly reduces serum diamine oxidase activity and serum D-lactate concentration. On the other hand, it enhances the mRNA expression of tight-junction proteins Occludin and ZO-1, improves the tight junctions in the duodenum, and further enhances intestinal barrier function [101]. This nutritional intervention not only helps maintain intestinal integrity, but also effectively alleviates intestinal inflammatory responses by regulating the Nrf2 and NF-κB signaling pathways [102]. In the study of Wassie et al. [10], it was found that supplementation of 400 mg/kg EP in the broiler diet significantly increased the VH and decreased the CD in the jejunum and ileum of broilers. Similarly, in the experiment by Sun et al. [103], it was found that supplementation with 3% EP significantly improved the nutrient utilization rate of the diet and the digestive enzyme activity in the intestines of broilers. In addition, dietary inclusion of 0.1% EP alleviates heat stress-induced tight-junction damage of intestinal epithelial cells in broilers by inhibiting the MLCK signaling pathway, reducing the phosphorylation modification level of MLC, and decreasing cytoskeleton sliding [73]. Therefore, the above observations indicate that EP improves the intestinal barrier function and nutrient absorption capacity, thereby playing a beneficial role in broiler production. Furthermore, molecular mechanism studies further reveal that marine sulfated polysaccharides (MSPs) can induce the expression of membrane-bound mucins (MUC1) and gel-forming mucins (MUC2 and MUC5AC), thereby promoting intestinal mucosal barrier function [104].

### 3.2. Beneficial Effects of Seaweed Active Substances in Laying Hens

#### 3.2.1. Improve Egg-Production Performance

Seaweed bioactive components consistently enhance laying-hen production performance through multiple nutritional regulatory mechanisms, including metabolic pathway modulation, oxidative stress alleviation, and microbial homeostasis maintenance (Table 4). As detailed in Table 4, the core advantages of red algae lie in lipid reconstruction and reproductive regulation; green algae excel in antioxidant effects and intestinal function optimization; brown algae can powerfully intervene in cholesterol metabolism; and cyanobacteria mainly focus on protein metabolism and structural strengthening. In poultry production, seaweeds should be selected based on their characteristics and practical needs. In the late laying period, hens suffer from reduced production performance and egg quality, due to oxidative stress accumulated from long-term egg laying. Supplementation with seaweed polysaccharides can significantly reduce the crack egg rate and increase the egg production rate, thereby improving the production performance of hens in the late laying period [105]. In general, seaweeds are rich in proteins, vitamins, minerals, and essential fatty acids, which are crucial for the overall health of laying hens. Supplementation with seaweeds and their extracts not only boosts egg production and quality, but also enhances the health status of laying hens, providing an efficient and sustainable solution for laying-hen farming.

#### 3.2.2. Improve Egg Quality

Seaweeds and their extracts can regulate egg quality through multiple levels, including the reconstruction of nutritional components, regulation of biomineralization, and modulation of metabolic pathways. Dietary supplementation with seaweeds has been shown to reduce cholesterol content in eggs, increase the levels of n-3 PUFA and iodine, and improve yolk coloration [94]. For example, adding 6% *Sargassum* powder to the diet of laying hens can increase the iodine content in eggs [113]. The addition of *Spirulina* can significantly increase the eggshell thickness and has a liver-protective effect on laying hens [112]. Supplementation with *Sargassum* extract (SL-CaNps) (up to 1.5 g/kg diet) not only increases the serum calcium and phosphorus concentrations, but also significantly improves eggshell thickness, eggshell weight per unit area, and the ultrastructure of eggshells [114]. Moreover, supplementation with 1% *Chondrus crispus* and 1% *Sarcodiotheca gaudichaudii* in the laying-hen diet can both increase yolk weight and egg weight [115]. Meanwhile, seaweed polysaccharides can synergistically improve yolk color and eggshell quality [105]. Therefore, seaweeds and their extracts can be used as potential nutritional regulators for egg quality. However, based on food safety considerations, attention should be paid to the accumulation of toxic and harmful substances in seaweeds [116], and safe and efficient application can be achieved through pretreatment.

## 4. Conclusions and Future Prospects

As a natural and effective feed additive, seaweed-derived active substances not only enhance poultry production performance, intestinal barrier function, disease resistance, and antioxidant capacity, but also improve the quality of meat and egg products. Furthermore, seaweed powder offers a potential alternative to traditional protein sources, thereby reducing the environmental pressure associated with livestock farming. Supplementing poultry diets with seaweed extracts also helps mitigate heat stress challenges during growth, which is significant for poultry health and sustainable production. However, the application of seaweeds and their active substances in poultry production still faces many challenges. For instance, high ash content, variable digestibility, and the potential for heavy-metal bioaccumulation in seaweeds can limit their practical use. In addition, while this review summarizes the primary mechanisms of seaweed actives in poultry involved in exerting anti-inflammatory, intestinal health-regulating, and antioxidant effects by regulating signaling pathways such as NF-κB, ERK, MAPKs, MLCK, and Nrf2, the specific molecular mechanisms of their actions need further in-depth study. Simultaneously, the structural diversity of seaweed actives from different sources and extraction methods is considerable, and systematic research on their structure–activity relationships is lacking. Therefore, ongoing research in this field remains imperative, to facilitate the utilization of seaweed resources in the poultry industry.

## Figures and Tables

**Table 1:** The chemical composition components among three types of seaweed.

Items	Lipid Content (%DW)	Carbohydrate Content (%DW)	Protein Content (%DW)	Dietary Fiber (%DW)	References
Brown algae	0.1–11.5	12.8–81	3.1–42.1	2.9–75	[27]
Green algae	0.2–15	4–79.9	0.4–32.1	1.1–64.9	
Red algae	0.4–12	8.3–68.2	3.5–47	3.3–65.6	

**Table 2:** The main polysaccharides and their biological activities in seaweeds.

Seaweed Species	Main Polysaccharides	Source of Polysaccharides	Composition Structure	Biological Activity	References
Red algae	Carrageenan	*Kappaphycus alvarezii* (Rhodophyta, Solieriaceae), etc.	A linear sulfated polysaccharide composed of D-galactopyranose linked by β-(1,3)- and α-(1,4)-glycosidic bonds.	Antiviral, anti-inflammatory, anticoagulant, lipid metabolism regulation, and hypoglycemic effects, etc.	[38]
	Agar	Gracilariaceae, Gelidiaceae, Pterocladiaceae, etc.	A long-chain neutral polysaccharide formed by the alternating repetition of β-D-Gal linked by 1,3-glycosidic bonds and 3,6-anhydro-α-Gal linked by 1,4-glycosidic bonds.		[39]
Brown algae	Fucoidan	*Fucus vesiculosus* (Ochrophyta, Fucaceae), etc.	A linear core (formed by (1→6)-β-d-Gal and/or (1→2)-β-d-Man units) with branched chains of “fucan” (formed by (1→3) and/or (1→4)-α-l-Fuc, (1→4)-α-d-GlcA, teminal β-d-Xyl and, sometimes, (1→4)-α-d-Glu).	Antithrombotic, antioxidant, hypolipidemic, and immunomodulatory effects, etc.	[33,40]
	Alginate	*Macrocystis pyrifera* (Ochrophyta, Laminariaceae), *Laminaria hyperborea* (Ochrophyta, Laminariaceae), *Ascophyllum nodosum* (Ochrophyta, Fucaceae), etc.	A linear polysaccharide composed of three forms: β-D-mannuronic acid (M residues), α-L-guluronic acid (G residues), or alternating M and G residues.		[28,41,42]
Green algae	Ulvan Polysaccharide	*Enteromorpha prolifera* (Chlorophyta, Ulvaceae), etc.	A water-soluble sulfated polysaccharide mainly composed of glucuronic acid, xylose, rhamnose, and galactose.	Immunomodulation, anti-inflammation, anti-tumor effects, etc.	[43,44]

**Table 3:** The application of seaweeds and their active substances in broiler production.

Seaweed Species	Seaweed	The Optimal Addition Amount of Seaweed (in the Basal Diet)	Mechanism of Action	Application Effectiveness	References
Red algae	*Lithothamnium calcareum* (Rhodophyta, Corallinaceae)	1018 μg/kg AFB1 + 0.2% *L. calcareum*	Binding of surface functional groups (such as carboxyl and hydroxyl) of *L. calcareum* to AFB1 for AFB1 adsorption.	Alleviation of AFB1-induced hepatic injury in broilers.	[83]
	*Kappaphycus alvarezii* (Rhodophyta, Solieriaceae)	1.0 g/kg seaweed extract PBD1	Intestinal gene expression controlling gut permeability and mucosal immunity.	Growth performance and immune response in broilers ↑.	[84]
	*Halymenia palmata* (Rhodophyta, Halymeniaceae)	0.25%	—	Linear growth trend of body weight gain and feed conversion rate.	[85]
	*Eucheuma denticulatum* (Rhodophyta, Solieriaceae)	1.0 g/kg seaweed extract PBD5	Enhancement of intestinal immunity and growth hormone-receptor gene expression.	Feed efficiency ↑, intestinal pathogen load↓, body growth ↑.	[77]
	*Chondrus crispus* (Rhodophyta, Gigartinaceae)	0.30%	—	Carcass and breast meat yield ↑, abdominal fat yield ↓	[86]
		0.40%	—	Relative weights of bursa and thymus ↑, and maintenance of intestinal pH stability.	
	*Chondracanthus chamissoi* (Rhodophyta, Gigartinaceae)	1.5%	Improvement in villus height (VH) and the ratio of villus height to crypt depth (VH:CD), and increase in the abundance of beneficial bacterial genera.	Intestinal morphology ↑, and growth performance ↑.	[87]
Brown algae	*Undaria pinnatifida* (Ochrophyta, Alariaceae)	0.5%	—	Weight ↑, mortality ↓.	[88]
	*Hizikia fusiformis* (Ochrophyta, Sargassaceae)	0.5%			
	*Ascophyllum nodosum* (Ochrophyta, Fucaceae)	2%	Reduction in plasma alanine aminotransferase (ALT) and gamma-glutamyl transferase (GGT) activities in broiler chickens.	Heat stress alleviation, and growth performance ↑.	[89]
Green algae	*Enteromorpha prolifera* (Chlorophyta, Ulvaceae)	0.1 mg/kg AFB1 + 0.25% *Enteromorpha prolifera* polysaccharides	Inhibition of phase I detoxification enzymes and up-regulation of the p38MAPK/NRF2–mediated phase II detoxification enzyme pathway.	Alleviation of AFB1-induced hepatic injury in broilers.	[90]
		100 mg/kg AFB1 + 2500 mg/kg *Enteromorpha prolifera* polysaccharides	Regulation of NRF2-mediated redox and mitochondrial apoptosis signaling pa.thways in broilers	Alleviation of AFB1-induced bursal injury in broilers.	[91]
		7000 mg/kg	Up-regulation of *Lipin1* and *SESN1* gene expression, down-regulation of *FGFR2* gene expression, promotion of protein synthesis and reduction in fat deposition.	Pectoral muscle yield ↑.	[92]
	*Ulva lactuca* (Chlorophyta, Ulvaceae)	Replacement of corn in the basal diet with 3.0% *U*. *lactuca*	—	Slaughter rate and pectoral muscle rate in broilers ↑.	[20]

↑: Improving; ↓: Decreasing.

**Table 4:** The application of seaweeds and their active substances in layer-hen production.

Seaweed Species	Seaweed	The Optimal Amount of Seaweed to Add (in the Basal Diet)	Mechanism of Action	Application Effectiveness	References
Red algae	*Kappaphycus alvarezii* (Rhodophyta, Solieriaceae)	1.5%	Reduction of lipid oxidation and cholesterol content of egg yolks.	Improvement in age at sexual maturity, egg production, egg quality and immune response.	[9,106]
	*Chondrus crispus* (Rhodophyta, Gigartinaceae)	3%	Stimulation of the growth of beneficial intestinal bacteria and inhibition of Salmonella colonization.	Weight ↑, feed conversion ratio (FCR) for egg production ↓, egg production rate ↑.	[107]
	*Chondrus crispus* (Rhodophyta, Gigartinaceae)	4%	Increased concentration of SCFAs in cecum contents and decreased Salmonella colonization.	Immune response ↑, improvement in intestinal microbial community structure, and growth performance and egg quality ↑.	[108]
	*Sarcodiotheca gaudichaudii* (Rhodophyta, Solieriaceae)	4%			
Green algae	*Caulerpa lentillifera* (Chlorophyta, Caulerpaceae)	1.5%	Increased protein intake, multiple antioxidant ingredients to scavenge free radicals in the body and reduce oxidative stress.	Maintenance of liver health, egg production rate ↑, and antioxidant activity in egg yolk ↑.	[109]
	*Ulva* sp. (Chlorophyta, Ulvaceae)	0.5~1.0% Ulvan	Up-regulation of intestinal FXR and hepatic CYP7A1 gene expression, reduction of cholesterol levels, and improvement in intestinal function.	Immune function ↑, antioxidant capacity ↑, improvement in laying performance and egg quality.	[110]
Brown algae	*Sargassum binderi* (Ochrophyta, Sargassaceae)	16%	Reduction of cholesterol content and low-density lipoprotein (LDL) in hen plasma and egg yolk.	Improvement in lipid metabolism in hens and egg quality ↑.	[111]
Cyanoba-cteria	*Spirulina platensis* (Cyanobacteria, Oscillatoriaceae)	3 kg/t	Reduction of cholesterol levels in serum and egg yolk.	Egg-laying performance ↑, egg quality ↑ and FCR ↑.	[112]

↑: Improving; ↓: Decreasing.

## Data Availability

Not applicable.
